# PHYSICAL STRAIN OF WALKING IN PEOPLE WITH NEUROMUSCULAR DISEASES IS HIGH AND RELATES TO STEP ACTIVITY IN DAILY LIFE

**DOI:** 10.2340/jrm.v56.40026

**Published:** 2024-06-07

**Authors:** Sander OORSCHOT, Eric L. VOORN, Annerieke C. VAN GROENESTIJN, Frans NOLLET, Merel A. BREHM

**Affiliations:** 1 Department of Rehabilitation Medicine, Amsterdam UMC location University of Amsterdam, Amsterdam, The Netherlands; 2 Amsterdam Movement Sciences, Rehabilitation & Development, Amsterdam, The Netherlands

**Keywords:** neuromuscular disorders, activities of daily living, physical exertion, exercise test, physical therapy

## Abstract

**Objective:**

To determine the physical strain of walking and assess its relationship with daily steps and intensity of daily activity in people with neuromuscular diseases.

**Design:**

Cross-sectional study.

**Subjects/patients:**

Sixty-one adults with neuromuscular diseases.

**Methods:**

Physical strain of walking, defined as oxygen consumption during comfortable walking relative to peak oxygen uptake. Daily step count and daily time spent in moderate and vigorous physical activity were assessed using accelerometry and heart rate measurements, respectively. Regression analyses assessed the relationships between log daily step count and log daily time spent in moderate and vigorous physical activity, and physical strain of walking.

**Results:**

The mean (standard deviation) physical strain of walking was 73 (20)% Log daily step count and physical strain were negatively associated (β = –0.47). No association was found with log daily time spent in moderate and vigorous physical activity.

**Conclusions:**

The highly increased physical strain of comfortable walking indicates that walking is very demanding for people with neuromuscular diseases and is associated with a reduction in daily step activity. The absence of a relationship between intensity of activities and physical strain indicates that, despite a reduction in daily step activity, strenuous daily activities may still be performed.

LAY ABSTRACT

The percentage at which comfortable walking is performed relative to a person’s exercise capacity is called the physical strain of walking. An increased physical strain of walking might reduce daily activity to preserve energy for other tasks. In this study, the physical strain of walking was determined in 61 adults with neuromuscular diseases, and we assessed the relationships between physical strain of walking and daily activity. We found that the physical strain of comfortable walking was highly increased, and walking is thus very demanding for people with neuromuscular diseases. Also, people with high physical strain took fewer steps. Understanding the profiles of daily activity and associated factors can guide rehabilitation interventions to address daily physical functioning. Future studies should investigate whether the physical strain of walking can be reduced by increasing the exercise capacity or reducing the energy required during walking, and analyse whether this improves daily activity.

Physical activity positively affects physical health, mental health, and quality of life and can reduce the risk of various chronic diseases (1). In people with neuromuscular diseases (NMD), physical activity is reduced in comparison with the general population (2, 3), due to muscle weakness (4), fatigue (5), and pain (6). Furthermore, disease severity (7), walking limitations (4), fear of falling (8), lower self-efficacy (9), and higher age and BMI (8) are reportedly associated with reduced physical activity in NMD. Yet, as shown in polio survivors, these factors, or combinations thereof, explained only 14–31% of the variance in physical activity (4, 8), suggesting the importance of other factors.

Based on findings in cerebral palsy (10), an elevated physical strain of walking might also be associated with reduced physical activity in NMD. Physical strain of walking is defined as the oxygen consumption of walking (VO_2walk_) relative to the peak oxygen uptake (VO_2peak_). In people with NMD, the VO_2peak_ has been shown to be up to 50% lower, compared with age- and sex-matched healthy individuals (11, 12), while the oxygen consumption of walking (VO_2walk_) is reportedly higher (13) due to deviations in the gait pattern (14, 15). A reduced VO_2peak_ together with an increased VO_2walk_ results in an elevated physical strain of walking. In studies among polio survivors, the physical strain of walking (16) andsubmaximal cycling (17) was found to be 65% and 75%, respectively, which is much higher than the physical strain of walking of 34–49% in able-bodied individuals (18, 19). Yet, the studies in polio survivors (16) used predicted maximal heart rate values instead of actual measured VO_2peak_ values, which is less accurate.

A high physical strain of walking may negatively impact daily physical activity (10). Physical activity is often measured by daily step count, which has been shown to be 30% lower in NMD compared with healthy individuals (20). The intensity of physical activity can be categorized according to the time spent in different intensity zones, whereby moderate to vigorous physical activity (MVPA) is recommended by health authorities (21) to reduce the risk of morbidity and mortality (22). Studies in NMD on the time spent in intensity zones reported inconsistent results. Studies mainly using questionnaires (7, 23) reported that people with NMD met the physical activity recommendations, whereas other studies, mostly using accelerometry, gave the opposite results (24–26). This inconsistency in results emphasizes the need for more studies objectively determining the time spent in intensity zones. Moreover, the physical strain of walking in NMD and the associations with both the quantity and intensity of daily activity are unknown, warranting study thereof in this population.

We aimed (*i*) to determine the physical strain of walking based on actual measured VO_2walk_ and VO_2peak_ in individuals with NMD, and (*ii*) to assess the relationship between daily physical activity, expressed as daily step count and time spent in MVPA, and physical strain of walking.

## METHODS

Data for this cross-sectional study were obtained from a randomized controlled trial, I’M FINE, investigating the efficacy of a physical activity programme for people with slowly progressive NMD (27). For this study, we used the data collected at baseline. The I’M FINE study was approved by the medical ethics committee of Amsterdam UMC, location Academic Medical Center (AMC), the Netherlands, and registered at the Dutch Trial register (NL7344). All subjects signed informed consent before study enrolment. Study reporting is in accordance with the ‘STrengthening the Reporting of OBservational studies in Epidemiology’ (STROBE) guidelines.

### Participants

Participants in the I’M FINE study were recruited between September 2018 and April 2022 from the departments of Rehabilitation Medicine of two university hospitals and four rehabilitation centres throughout the Netherlands, and through the nationwide Dutch patient organization for NMD (Spierziekten Nederland). Potentially eligible individuals received an information letter. After a phone call to assess general inclusion criteria, individuals willing to participate were invited for a screening visit by a rehabilitation physician. Main inclusion criteria were: diagnosed with Charcot-Marie-Tooth disease, post-polio syndrome, or other slowly progressive NMD; aged ≥ 18 years; and motivated to follow a physical activity programme to improve aerobic capacity. Participants were excluded if they had contraindications for physical activity according to the American College of Sports Medicine (ACSM) guidelines (28); were unable to follow verbal or written instructions; had insufficient understanding of the Dutch language; or had participated in an exercise programme for more than 4 weeks in the past 6 months (27). Only ambulatory participants with complete and valid baseline data for all variables of interest, as defined below, were included in the analyses of the current study.

### Measurements

Measurements were performed at the Department of Rehabilitation Medicine of the AMC. First, participant characteristics and demographics were registered. Furthermore, muscle strength of 8 muscle groups of both legs (hip flexion/extension, hip abduction/adduction, knee flexion/extension, ankle plantar/dorsal flexion) was determined by manual muscle testing, and scored according to the Medical Research Council (MRC) scale (29). Scores of all muscle groups were summed to calculate a composite muscle strength score, referred to as MRC sum score (range 0–80). Subsequently, participants performed a walking energy cost test (WECT) at comfortable speed to assess VO_2walk_ and walking speed, followed by a maximal incremental exercise test to assess VO_2peak_. In addition, daily physical activity during waking hours was measured for 7 consecutive days.

*Physical strain of walking.* The physical strain of walking (in % of VO_2peak_) was calculated as the ratio of VO_2walk_ (measured during the WECT) and VO_2peak_ (measured during the maximal incremental exercise test), multiplied by 100%.

*Oxygen consumption of walking (VO*_2walk_*).* After an 8-minute rest period, participants performed a 6-minute WECT at self-selected, comfortable speed on a 30-meter indoor track. The use of assistive devices (e.g., cane, stick, walker) during the test was allowed. Throughout the test, breath-by-breath oxygen consumption was measured with a portable gas analysis system (K5 Gas Analysis System, Cosmed, Rome, Italy). Before every test, the gas analysis system was calibrated according to the manual instructions. We visually determined a steady state period of at least 60 seconds between the third and sixth minute of the test and calculated the mean oxygen consumption (VO_2walk,_ in ml/kg/min) over this steady-state period. Walking speed (in m/s) was calculated as the total distance walked divided by 360 seconds. Previously, the WECT with simultaneous breath-by-breath gas analysis has been shown to be valid for measuring VO_2walk_ in people with NMD (16).

*Peak oxygen uptake (VO*_2peak_*).* After screening for contraindications by a physician, a maximal incremental exercise test on a bicycle ergometer (Lode Excalibur, Lode BV, Groningen, The Netherlands) was executed according to international guidelines concerning standardization, and supervised by a trained researcher (28). After 3 minutes of rest, the test started with 3 minutes unloaded cycling, followed by 5–20 Watt/minute continuous workload increments, depending on the participants’ physical fitness level. During the test, oxygen uptake was continuously measured using MasterScreen CPX (CareFusion, Hoechberg, Germany) (30). The test was stopped if 1 of the following criteria was met: oxygen uptake plateau reached, pedal frequency dropping below 50 revolutions per minute, or the participant met the ACSM stop criteria for exercise testing (28). VO_2peak_ (in ml/kg/min) was determined as the highest 30 s moving average.

*Daily physical activity.* Daily physical activity was determined by step count and time spent in MVPA. Participants were provided with an accelerometer (ActiGraph GT3X+ accelerometer, Health One Technology, Fort Walton Beach, FL, USA) and a heart rate belt (Polar H10, Polar Electro, Kempele, Finland). They were asked to wear the accelerometer on the left side of their waist and the heart rate belt just below their sternum for all waking hours (except for bathing and swimming) for 7 consecutive days (27). A demonstration was given on proper positioning of both devices and participants were provided with written instructions on placement and wear time of the device. The accelerometer and heart rate belt were connected and the data were analysed with the ActiLife software (version 6.13.4, Health One Technology, Fort Walton Beach, FL, USA). Physical activity data were used if at least 4 days of 480 minutes or more were measured. From the measured data we calculated the mean daily step count. Intensity zones were individually calculated based on percentages of the heart rate reserve (HRR), defined as the peak heart rate during the maximal incremental exercise test minus the resting heart rate. Intensity zones were categorized as low (< 40% HRR), moderate (40–60% HRR), and vigorous (> 60% HRR) (22). For this study, we summed the daily time spent in the moderate and vigorous activity zones as time spent in MVPA.

### Statistical analysis

Descriptive data were expressed as mean and standard deviation (SD) in the case of normally distributed data, or as median and interquartile range (IQR) if data were not normally distributed, based on visual inspection. To determine whether the physical strain of walking was increased in our sample of subjects with NMD, we compared the outcome with previously published reference data of healthy subjects of similar age (18, 19).

Relationships between daily step count and daily time spent in MVPA (dependent variables) with physical strain (independent variable) were assessed with linear regression analyses (2 separate models). If the dependent variables were not normally distributed, they were transformed using the natural-log function to approximate a normal distribution. Regression analyses were checked for confounding effects of gender, age, body mass index (BMI), MRC sum score of lower extremities, and comfortable walking speed. Each potential confounder was entered separately into the uncorrected model and the strongest confounder that changed the ß by at least 10% was added to the model. This procedure was repeated until there were no more confounders to add to the corrected model. To explore effect modification, we added the interaction of muscle strength (MRC sum score), which we considered to be the most plausible effect modifier, to the corrected models, and reported whether it was an effect modifier or not. All analyses were performed using SPSS (version 26.0, IBM Corp, Armonk, NY, USA). A *p-*value of < 0.05 was considered statistically significant.

## RESULTS

Of the 91 subjects included in the I’M FINE study, 61 had complete and valid baseline data for all variables of interest. Incomplete or invalid data were due to missing or incomplete daily activity data (*n* = 23), invalid VO_2walk_ determination (*n* = 5), or non-ambulatory participants (*n* = 2). There were no significant differences in subject characteristics between the groups included (*n* = 61) and excluded (*n* = 30) for the analyses.

**Table I:** Characteristics of subjects

Sex (male/female), *n*	24/37
Age (years), median (IQR range)	63.0 (50.5–67.0)
Height (cm), mean (SD)	171.7 (10.5)
Weight (kg), mean (SD)	75.8 (14.2)
BMI, mean (SD)	25.8 (4.9)
MRC sum lower extremities (range: 0–80), median (IQR range)	73.3 (63.2–76.8)
Walking aid (yes/no), *n*	45/16
Diagnosis (PPS/CMT/other), *n*	14/31/16

BMI: body mass index, CMT: Charcot-Marie-Tooth disease, MRC: Medical Research Council scale, PPS: post-polio syndrome.

Subjects (37 females) were diagnosed with Charcot-Marie-Tooth disease (*n* = 31), post-polio syndrome (*n* = 14), or other NMD (*n* = 16) (Table II). Participants with “other NMD” were diagnosed with congenital myopathy (*n* = 5), limb-girdle muscular dystrophy (*n* = 4), myotonic dystrophy (*n* = 3), inclusion body myositis (*n* = 2), oculopharyngeal muscular dystrophy (*n* = 1), and chronic idiopathic axonal polyneuropathy (*n* = 1). We observed MRC sum scores below 80 in 54 participants (89%). The median (IQR) MRC sum score was 73.3 (63.2–76.8).

**Table II:** Physical strain of walking and daily activity outcomes

Physical strain of walking (%), mean (SD)	72.6 (20.3)
VO_2walk_ (ml/min/kg), mean (SD)	14.9 (2.9)
VO_2peak_ (ml/min/kg), mean (SD)	21.7 (7.8)
Walking speed (m/s), mean (SD)	1.0 (0.2)
Daily step count, median (IQR range)	4563 (3567–6664)
Time spent in MVPA (min per day), median (IQR range)	55 (25–109)

ml: millilitre, min: minute, kg: kilogram, m: meter, s: second, h: hour, MVPA: moderate and vigorous physical activity zones.

The mean (SD) physical strain of walking was 73 (20)%, and 34–49% in control groups of able-bodied individuals of similar age (18, 19) (Table II). The median (IQR) daily step count was 4,563 (3,567–6,664) steps, and 55 (25–109) minutes spent daily in MVPA.

Daily step count and time spent in MVPA were both not normally distributed and these variables were therefore transformed using the natural-log function. We found a significant association between log daily step count and physical strain of walking, in both the corrected and uncorrected regression analyses (Table III). The corrected regression analysis showed an inverse relationship (β = –0.47) between log daily step count and physical strain of walking. No significant association between log daily time spent in MVPA and physical strain of walking was found (Table III). There were no significant interactions between lower extremity MRC sum score and the independent variables in the corrected analyses.

**Table III:** Linear regression analyses with log daily step count and log time spent in MVPA as dependent variables and physical strain of walking as independent variable

Factor	Regression coefficient (β)	95%-CI	*p*-value
Log daily step count and physical strain (uncorrected)	–0.58	–0.79; –0.37	< 0.001
Log daily step count and physical strain (corrected)^a^	–0.47	–0.67; –0.27	< 0.001
Log time spent in MVPA and physical strain (uncorrected)	0.21	–0.05; 0.46	0.11
Log time spent in MVPA and physical strain (corrected)^b^	0.12	–0.15; 0.40	0.37

a Corrected for age, walking speed.

b Corrected for gender, age, body mass index.

CI: confidence interval, MVPA: moderate to vigorous physical activity.

## DISCUSSION

Our study in ambulatory people with different NMDs showed that physical strain of comfortable walking was severely increased, indicating that walking is highly demanding. Log daily step count was significantly inversely related to physical strain of walking, while log time spent in MVPA was not related to physical strain of walking.

The mean physical strain of walking of 73% we found is comparable to the physical strain of walking of 65% reported in polio survivors, and much higher than the reportedly 34–49% in able-bodied individuals of similar age (18, 19). It is also considerably higher than the physical strain of 50–52% found in other patient groups, including children and adults with cerebral palsy (10, 31), and adults post-stroke (18). The results from our study and previous studies imply that walking for people with NMD is highly demanding, and this may negatively influence health, daily life activities, and quality of life (32, 33).

A low VO_2peak_ rather than a high VO_2walk_ seemed to be the main contributor to the high physical strain of walking in our sample. Comparable to previous studies in NMD (11), we found a VO_2peak_ of 21.7 ml/min/kg, which is on average 30–44% lower than the VO_2peak_ reported in healthy subjects of similar age (34), possibly caused by the reduced muscle mass due to the progressing muscle weakness in NMD (17). The VO_2walk_ in our sample was not increased and comparable to values found in healthy subjects of similar age (18, 19). This likely resulted from the 20–26% reduction in walking speed in our subjects with NMD, as compared with healthy subjects (18, 19). The latter finding is in line with the energy minimization theory (35) and earlier research in NMD (36), showing that people with NMD walk slower to prevent increases in the oxygen consumption of walking, so that it can be sustained for longer periods in daily life. To lower the high physical strain of walking in people with NMD, neuromuscular rehabilitation could focus on increasing the VO_2peak_ by means of aerobic exercise. Based on a recent systematic review on aerobic capacity in NMD (11), this may be especially beneficial in people with lower baseline VO_2peak_ levels compared with those with higher baseline VO_2peak_ levels. Another approach to reduce the physical strain of walking could be to the decrease the VO_2walk_ in those people with an elevated VO_2walk_ and/or to increase walking speed, without affecting the VO_2walk_, for example by prescribing or optimizing lower extremity orthoses (37).

The impact of a high physical strain of walking on daily activity in our subjects with NMD was highlighted by the inverse relation with the log daily step count, i.e., people with a high physical strain of walking took fewer steps per day. This is in line with the inverse relation between physical strain of walking and total daily walking time reported in adults with cerebral palsy (10). However, no relationship was found between physical strain of walking and the log daily time spent in MVPA in our study. This may indicate that, despite a reduction in daily step activity, strenuous daily activities may still be performed. An earlier study on Charcot-Marie-Tooth disease (25) also found a lower daily step count, but no differences in time spent in MVPA, as compared with healthy subjects. As suggested by the authors, the difference between the 2 daily activity outcomes could be due to people breaking up sedentary periods with more frequent transitions into standing, without accumulating a larger number of steps. In line with their recommendation, more detailed exploration with accelerometers that can distinguish between different activities, such as lying, sitting, and standing, would be required. Furthermore, they suggested an overestimation of the time spent in MVPA as the activity monitor used was not validated in the NMD population and did not correct for potential reduced energy expenditure due to reduced muscle mass. However, this suggestion seems unlikely as we found similar results while using individually determined time spent in MVPA, based on heart rate measurements. Alternatively, in line with Horemans et al. (33), it should be realized that intensity of daily-life activities is not only determined by walking ability but also by other factors, such as social behaviour, personal lifestyle, working conditions, and living circumstances. Moreover, certain tasks have to be done in daily life, despite high intensity or strain.

### Limitations

First, we used baseline data of an intervention study for people with NMD who were motivated to increase their aerobic capacity. Moreover, all participants were ambulant, limiting generalizability to other, for instance non-ambulant, people with NMD. It would be interesting for future research to investigate the relationship between the physical strain of wheelchair use and its relationship with daily activity. Second, while we were interested in the physical strain of walking, the VO_2peak_ was determined during cycling and not during walking. As the measured VO_2peak_ during walking is generally higher than the VO_2peak_ during cycling (38), due to a larger exercising muscle mass during walking, this may have led to an overestimation of the physical strain of walking. Finally, although the accelerometer we used to determine steps has often been used in earlier studies assessing daily-life physical activity with proven good validity for assessing daily step count in different elderly populations, its validity to assess step count at lower walking speeds (< 0.9 m/s) is low (39). Some of our subjects had a walking speed lower than 0.9 m/s, which could have influenced the investigated relation between the physical strain of walking and daily steps taken.

### Conclusion

Our study showed that the physical strain of comfortable walking was severely increased. On average the physical strain was 73%, indicating that walking is highly demanding in people with NMD. The number of daily steps taken was inversely related to the physical strain of comfortable walking, while intensity of daily activity was not related. Thus, people with increased physical strain took fewer steps, but the absence of a relationship with moderate and vigorous intensive activities may indicate that, despite a reduction in daily step activity, strenuous daily activities may still be performed. Reducing the physical strain of walking by increasing the VO_2peak_ with aerobic exercise or by reducing the VO_2walk_ by means of lower limb orthoses should be further investigated as potential clinical directions to improve daily activity in people with NMD, and studies with a longitudinal design should focus on the causal relation between physical strain of walking and daily activity.
